# In-Line and Off-Line Monitoring of Skin Penetration Profiles Using Confocal Raman Spectroscopy

**DOI:** 10.3390/pharmaceutics13010067

**Published:** 2021-01-07

**Authors:** Richard Krombholz, Yali Liu, Dominique Jasmin Lunter

**Affiliations:** Department of Pharmaceutical Technology, Eberhard Karls University, Auf der Morgenstelle 8, 72076 Tuebingen, Germany; richard-paul.krombholz@uni-tuebingen.de (R.K.); cpuyali@gmail.com (Y.L.)

**Keywords:** in-line and off-line measurements, confocal Raman spectroscopy, procaine-HCl penetration, PEG-23 lauryl ether, penetration enhancement, skin

## Abstract

Ex-vivo and in-vivo skin analysis has been extensively evaluated by confocal Raman spectroscopy (CRS). The off-line measurement with a CRS-suited skin-mounted device after Franz-cell incubations is the most popular choice. However, real-time monitoring of in-line measurement has clear advantages for obtaining dynamic and more timely results. In our study, a custom-built setup suitable for in-line measurements was implemented, which ensures constant skin incubation and in-situ skin detections. We aim to compare the differences between using in-line and off-line devices for monitoring skin drug penetrations. A well-assessed formulation gel with procaine-HCl as the active ingredient was used as reference. The PEG-23 lauryl ether was added to the formulation as a penetration enhancer to evaluate the enhancement effects of procaine on skin. After incubation times of 14, 20, and 24 h, skin penetration profiles were assessed. Comparable results between off-line and in-line measurements were obtained. Remarkable improvements in penetrated procaine amount and depth were observed. Based on the significant differences of their enhanced penetration amounts, fairly similar estimations were achieved from both methods. A slight difference of 14 h incubation between these two setups can still be found, which may be due to the different detection conditions and affected skin properties. Overall, in-line measurements could provide a more time- and labor-saving alternative for off-line measurements in ex-vivo study.

## 1. Introduction

The skin is the largest organ of the human body and is considered as a crucial route for drug deliveries [1,2,3]. With the topical application of components, non-invasive and efficient techniques for monitoring their skin penetrations are strongly needed.

Nowadays, confocal Raman spectroscopy (CRS) as a method for skin penetration studies in-vivo and ex-vivo is on the rise [4,5,6,7]. Different methods have been described for obtaining depth profiles of topically applied actives using CRS [8,9]. In skin in-vivo studies, in-line measurements have been widely described for monitoring the distribution of actives or excipients on human skin [10,11]. However, the use of human skin faces a lot of difficulties concerning accessibility due to ethnic problems and poor convenience. Thus, excised substitute such as porcine skin has been commonly used as an appropriate model [12,13]. Meanwhile, ex-vivo experiments play a very important role in the development of semi-solid dosage forms, not only as drug delivery systems but also in cosmetics. While in such ex-vivo skin penetration studies, in-line measurement is fairly new and rarely performed yet due to the complexity of producing the Raman device-suited skin incubation cells, although it offers the great benefits of timesaving and real-time monitoring [14,15,16].

Franz diffusion cells are the most commonly used method for skin penetration studies. The formulation to test is applied on either the excised human skin or a suitable surrogate, fixed between two compartments, and tempered to the human skin temperature of 32 °C. After a certain incubation time, the amount of drug in different skin layers or depths can be quantified. Either in a destructive way, by segmenting the skin, for example, with adhesive tape strips or by cryo-segmentation [17,18], followed by a quantification step, where high-performance liquid chromatography is the most popular method [19,20], or with a non-invasive method, as, for example, CRS [4,21,22]. Among other optical methods, as attenuated total reflectance infrared spectroscopy [23,24,25] or fluorescence microscopy [26,27], CRS has the advantage of high spatial resolution and chemical sensitivity, even when used for lateral depth scanning, making it an ideal technique for skin penetration studies. In-situ measurements have the further advantage of being even less time-consuming, as there is no separate incubation step required.

In our study, a suitable device for skin incubation and simultaneous CRS detection was developed and used for the in-line measurements. The device was custom-built in our department and has recently been described in [28]. The conventional stepwise CRS measurements with the stopping of the skin incubations in Franz cells were seen as the off-line method. The aim of this study was to evaluate differences in skin penetration profiles between off-line and in-line measurements. The procaine-HCl incorporated gel formulations were applied with and without penetration enhancer of PEG-23 lauryl ether (L23) on skin followed by the CRS detections. L23 contained an average number of 23 oxyethylene groups, has higher solubility in water and can be used in gel formulations. L23 was chosen as the penetration enhancer in our study as Shin et al., showed that it enhanced the penetration of local anesthetics. The calculated enhancement ratio has been observed with the incorporation of L23. The enhanced mechanism of this type of PEGylated emulsifiers might be induced by the disruption of stratum corneum lipid matrix and increased fluidity of lipid structures.

The formulations used in this study were chosen because their penetration rate and extent are already described [7], making them ideal for investigating the effect of the method used for skin penetration studies.

With comparisons of their penetration profiles, either the correlation of these two measurements can be established, proving their feasibility in use for future studies, or the respective details responsible for their differences can be addressed.

## 2. Materials and Methods

### 2.1. Materials

The model drug used was procaine-HCl, obtained from Ceasar & Loretz GmbH, (D-Hilden, Germany). PEG-23 lauryl ether was purchased from Croda GmbH, (Nettetal, Germany). Hydroxypropyl methylcellulose (HPMC) was from Shin Etsu Chemical Co. Ltd., (J-Tokyo, Japan). Polyoxyethylene polyoxypropylene copolymer (Poloxamer 407) was obtained from (BASF SE, D-Ludwigshafen, Germany). Parafilm^®^ was from Bemis Company Inc., (Oshkosh, WI, USA). Sodium chloride, disodium hydrogen phosphate, potassium dihydrogen phosphate, and potassium chloride were of European Pharmacopoeia grade. All aqueous solutions were prepared with ultra-pure water (Elga Maxima, High Wycombe, UK).

Porcine ear skins (German land race; age: 15 to 30 weeks; weight: 40 to 65 kg) were obtained from the Department of Experimental Medicine of the University Hospital Tuebingen. The live animals used were kept at the Department of Experimental Medicine and sacrificed in the course of the experiments, which were approved by the ethics committee of the University Hospital Tuebingen. The ears were obtained directly after the death of the animals. Prior to the study start, the Department of Pharmaceutical Technology was registered for the use of animal products at the District Office of Tuebingen (registration number: DE 08 416 1052 21).

### 2.2. Preparation of Gel Formulations

Procaine-HCl contained in a gel formulation was prepared to detect the procaine penetration on skin. The HPMC poloxamer gels were prepared according to the method published by Shin et al. [29] and have also been described in our previous studies [5,7]. The formulation without penetration enhancer contained 1 g of procaine-HCl, 0.2 g of HPMC, and 2 g of poloxamer 407 in 6.8 g water. The formulation with enhancer additionally contained 0.5 g PEG-23 lauryl ether (L23). The water content was thus reduced to 6.3 g. The ingredients of the formulation are described in detail in Table 1.

### 2.3. Preparation of Porcine Ear Skin

The donor pigs were 15 to 30 weeks old and weighed 40 to 65 kg. Fresh porcine ears were cleaned with isotonic saline. Full-thickness skin was removed from cartilage and gently cleaned from blood with cotton swabs and isotonic saline. The obtained postauricular skin sheets were then dried with soft tissue, wrapped with aluminum foil and stored in a freezer at −28 °C.

On the day of experiment, skin sheets were thawed to room temperature on PBS-soaked tissues, to prevent the skin from drying out, cut into strips of approximately 5 cm width and stretched onto a Styrofoam plate (wrapped with aluminum foil) with pins. Subsequently, skin hair was trimmed and the skin was dermatomed to a thickness of 1.0 mm (Dermatom GA 630, Aesculap AG & Co. KG, Tuttlingen, Germany). Then, the dermatomed skin used for Franz cell incubation was punched out for circles to a diameter of 25 mm whereas that used for in-situ incubation was cut out to a diameter of 35 mm. The procedures have been described in detail in previous publications [28,30].

### 2.4. Incubation of Dermatomed Porcine Ear Skin

Franz diffusion cell (Gauer Glas, Püttlingen, Germany), as a gold standard in ex-vivo dermal drug delivery and skin penetration and permeation experiments, was used as a benchmark here. The obtained data of drug penetrations can be compared with a recently established in-situ method [28] for detecting the drug penetration profiles by CRS (WITec GmbH, Ulm, Germany). After the Franz cell incubation for a certain time, skin samples were obtained and transferred to a specially made skin-placed device (mechanical workshop of the Institute for Pharmaceutical Sciences and the electronic workshop of the Institute of Chemistry, Tuebingen, Germany) for CRS measurements [5]. Thus, the incubation was stopped, which was deemed as the off-line method in this study. In contrast, dynamic measurements on the same skin samples for different incubation times can be carried out by using the custom-built in-line device.

**Figure 1:**
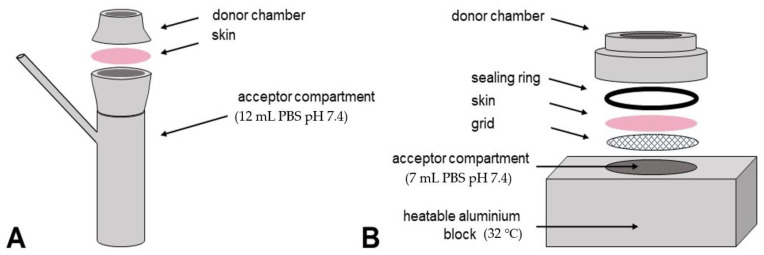
The schematic profiles of the off-line device (**A**) of Franz diffusion cell for skin incubation and in-line device (**B**) of skin incubation cells used.

For the incubation of skin samples in Franz-cells, 12 mL of degassed, prewarmed (32 °C) phosphate-buffered saline (PBS) was used as receptor fluid with a string speed of 500 rpm. Then, a skin sample was mounted and tightened with the donor compartment on top. Formulation prepared ahead with an amount of 0.25 g/cm^2^ was then applied, with a piece of parafilm used as a cover to prevent water evaporation. The unjacketed Franz-cells were placed in a 32 °C water bath for the incubation step. After incubation for 14, 20 and 24 h separately, skin samples were removed from cells and each skin surface was gently washed and cleaned with isotonic saline and cotton swabs to remove the remaining samples and avoid erroneous measuring results [5]. Then, the actual application area (15 mm in diameter) was punched out, patted dry with cotton swabs and mounted onto the device for CRS measurements (Figure 1A). For a detailed description of the exact procedures including the Franz-cell incubation and aforementioned skin-mounted device, please see our previous publications [5,31].

The set-up of the custom-built device for in-line measurements is shown in Figure 1. For the in-line skin incubation and CRS measurements, 7.0 mL of PBS was filled in the acceptor compartment and a skin sample was placed on the grid above, which was soaked in PBS previously to displace all air in the grid. After an equilibration time of 30 min to temper the skin and the device (32 °C), 2.0 mL of the formulation to test was applied (0.25 g/cm^2^). Parafilm was tightened between the objective of the Raman Microscope and the donor chamber to prevent any water loss. The schematic view of the custom-built in-line device is shown in Figure 1B. A detailed description of it can be found in our previous publication [28].

### 2.5. Confocal Raman Spectroscopy Analysis of Skin Penetrations

In-line and off-line devices used on CRS for detecting skin drug penetration profiles were compared. For both skin-placed devices, the skin spectra were acquired with an alpha 500R confocal Raman microscope (WITec GmbH, Ulm, Germany), which was equipped with a 532 nm excitation laser, UHTS 300 spectrometer and DV401-BV CCD camera. The optical grating was 1800 g/mm for recording the spectra in the range of 700 to 1800 cm^−1^. Laser power was adjusted to 25 mW for the off-line skin measurements and to 32 mW for the in-line measurements by the optical power meter (PM100D, Thorlabs GmbH, Dachau, Germany). Simultaneously, 63 × 1.0NA water immersion objective (W Plan-Apochromat, Carl Zeiss, Jena, Germany) was utilized, which also enabled the dynamic measurements of in-line device by dipping the immersing objective in the formulation gel.

**Figure 2:**
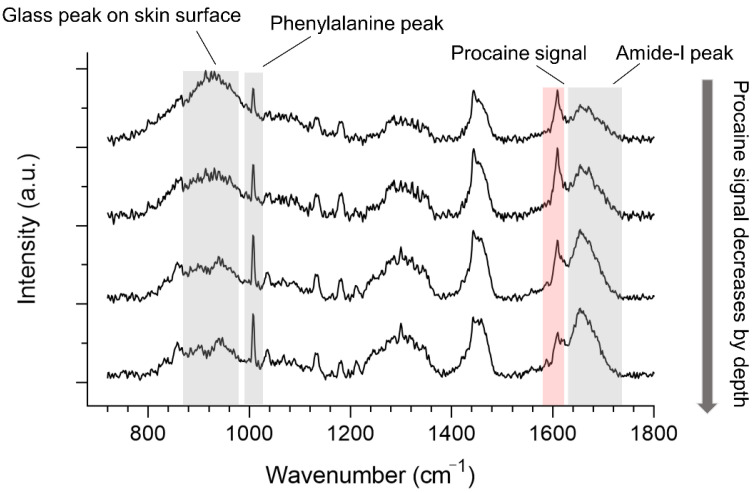
Typical spectra in off-line skin analysis of procaine signal decreasing by depth with crucial spectral features highlighted in colors.

In off-line analysis, a line scan was implemented in order to collect the skin spectra from depth, with the laser spot recording from 10 μm above the skin down to 40 μm inside the skin with a step size of 1 μm. The exposure time was 2 s for one measurement with 2 accumulations for each spectrum. Spectra were collected from three randomly selected spots on three skin samples, resulting in nine measurements. The skin pieces for in-line and off-line CRS measurements were from two different donors. Their comparison of penetration profiles was accomplished by analyzing the relative penetration enhancements from different incubation times. Furthermore, in the off-line spectral analysis, line scans were performed for quick collections of depth skin signals due to the non-stopping penetration behaviors inside the skin samples after taking them out of the incubation cells. Thus, the spatial resolution recorded by the line scan would appear to have a relatively lower resolution (Figure 2).

For in-line measurements, two-dimensional image scans of 5 µm width and 25 µm depth were performed, acquiring 10 spectra per line and 50 lines per vertical dimension, using an exposure time of 1.5 s per spectra. Measurements were performed after 14, 20 and 24 h, focusing always on the same position, chosen randomly at the beginning. Each formulation was tested on three pieces of skin of the same donor-animal on three different days. Three depth profiles out of every two-dimensional scan were extracted, resulting in 9 depth profiles per formulation (*n* ≥ 9).

The skin surface can be determined from different signal profiles by depth. The half maximum of the signal profile of the skin (amide-I, phenylalanine peak) and glass peak signals are both suitable for use. The skin surface in in-line measurements was determined as the position with the half maximal intensity of the phenylalanine signal. For off-line measurements, the phenylalanine signal will be affected and covered by the glass signal near the skin surface. Therefore, the skin surface here was determined as the position of the intersection of the amide-I and glass signals (Figure 2).

### 2.6. Spectral Data Analysis

The initial processing step of Raman spectra included the spectral cosmic ray removal, smoothing as well as background subtraction, which were performed using the WITec Project Software (Project 5 plus, WITec GmbH, Ulm, Germany). Referring to the smoothing process, a Savitzky-Golay (SG) filter was applied with a third polynomial order and nine smoothing points. For the background subtraction, an automatic polynomial function was fitted to the spectrum and subtracted. Furthermore, the area under the curve (AUC) in this study is the integrated area under a specified peak of the spectrum and could be calculated using a trapezoidal method on WITec Project Software.

### 2.7. Characterization of Penetration Profiles

Based on the spectral information, peak areas of the procaine peak and the amide-I peak were calculated using off-line measurements. The amide-I peak signal has been proved to be stable from different donors and within one donor and can be successfully used to normalize the procaine peak to account for the depth attenuation effects [30]. Thus, the relative procaine penetration in skin can be calculated by the ratio of AUC of the procaine and amide-I peaks.

**Figure 3:**
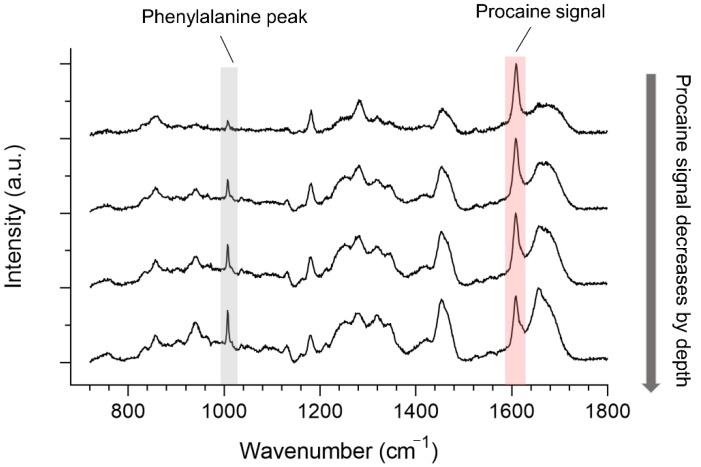
Typical spectra in in-line skin analysis of procaine signal decreasing by depth with crucial spectral features, which are highlighted in color.

Regarding the in-line measurements, depth penetration profiles were determined by calculating the AUC of the procaine peak and normalized to the arithmetic mean of the aromatic amino acid peak at 1008 cm^−1^, as this peak does not interfere with the spectra of procaine and because no coverslip was used, this peak does not overlap with any glass-signal (Figure 3), as with the off-line-measurements.

### 2.8. Statistical Data Analysis

Spectra were obtained from repeated measurements (*n* ≥ 9). The graphs were shown with mean values ± standard deviations (mean ± SD). Statistical differences were determined using one-way analysis of variance (ANOVA) followed by Student-Newman-Keuls (SNK), which were employed using GraphPad Prism 8.0 (GraphPad Software Inc., La Jolla, CA, USA). Diagrams and statistical differences were ultimately generated. Significant differences were marked with a different number of asterisks: * *p* < 0.05, ** *p* < 0.01, *** *p* < 0.001.

## 3. Results and Discussion

### 3.1. Off-Line Monitoring of Drug Penetration

The drug penetration of procaine-HCl out of two different hydrogel formulations was investigated from different time points, in order to display the effect of L23 as a penetration enhancer and to compare the obtained results to in-line measurements performed in parallel.

**Figure 4:**
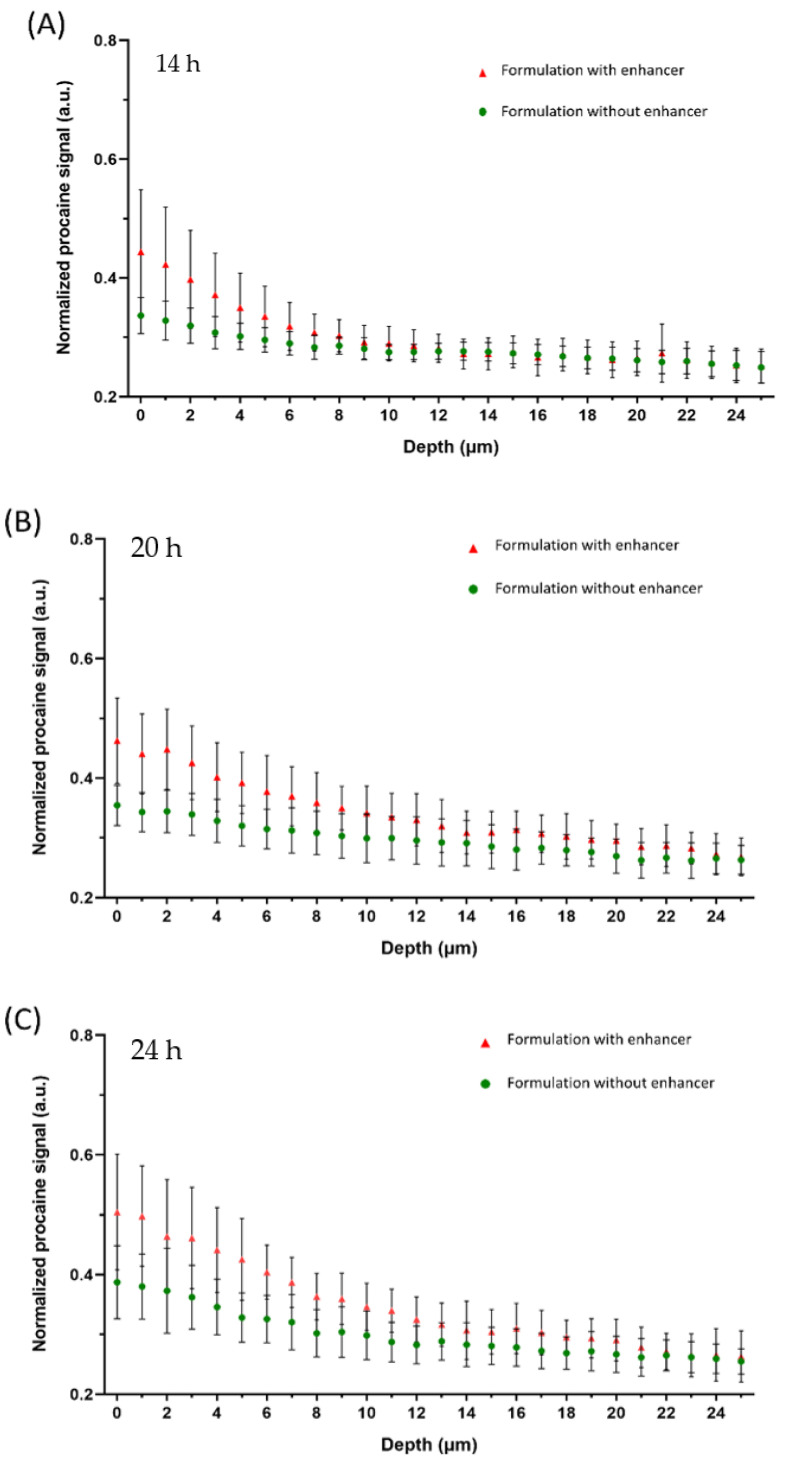
Penetration profiles of procaine HCl in porcine skin after the skin incubation time of 14 h (**A**), 20 h (**B**) and 24 h (**C**) with the measurements of the off-line device. Two procaine-HCl containing formulations were compared: formulation with enhancer (red triangles) and without enhancer (green circles). Mean ± SD (*n* ≥ 9).

Figure 4 shows the penetration profiles at different incubation times with and without the addition of L23 as the penetration enhancer.

Figure 4A shows the procaine penetrations after 14 h incubation within formulation gels. The depth detections reached more than 25 µm of the skin. The Raman intensity of the procaine band at 1612 cm^−1^ is displayed as a function of depth, where 0 is assigned to the surface of the skin. The red triangles represent the penetration profile of procaine out of the formulation containing L23 as a penetration enhancer, while the green circles show the penetration profile of the reference formulation without penetration enhancer. It is clear that the procaine signal was detected from the skin surface to around 6 µm of the skin with no enhancers added. However, procaine signals deeper in the skin of nearly 8–9 µm can still be found with the effect of adding L23 in formulations. This is in line with our previous studies [6,32]. Higher procaine signals were observed at the upper layer of the skin (within 4 µm). Thus, it becomes obvious that the addition of L23 enhanced the depth of drug penetration. These enhancement effects can be noticed, although in a shorter incubation time of 14 h.

Extending the skin incubation time to 20 h (Figure 4B), a higher amount of procaine in the skin could be detected for both formulations and the enhancement effect is even clearer. In detail, the reference formulation shows a higher penetrated amount of procaine in the upper layer of the skin compared with 14 h incubation. Meanwhile, the penetration depth presented a slight increase as well, to around 10 µm. Comparatively, in the formulation with the addition of L23, it can be better noticed that the enhancer strongly improved the procaine penetration to around 20 µm. The difference between the two formulations was more pronounced with 20 h skin incubations.

At the 24 h time point, Figure 4C shows a very similar tendency with 20 h incubation. However, the gap in their penetration profiles is more distinct than at 20 h. It can be noticed that the reference represented a similar penetration depth of procaine compared with 20 h incubation, with a detectable procaine signal to approximately 11 µm. While the penetrated amount is noticeably higher, especially within the first 5 µm of the skin. Likewise, the L23 added formulation indicated similar tendencies. The penetration depth of procaine with the enhancement effect of L23 was not more pronounced than after 20 h. A similar depth was remarked to be approximately 20 µm as the result of 20 h incubations.

Overall, time-dependent penetration properties were observed in the comparisons of two formulations. With the increase of incubation time, drug penetration amount and depth both showed apparent improvement. However, the maximum penetration depth was reached after around 20 h incubations. The penetrated drug amount constantly increased.

### 3.2. In-Line Monitoring Drug Penetration

**Figure 5:**
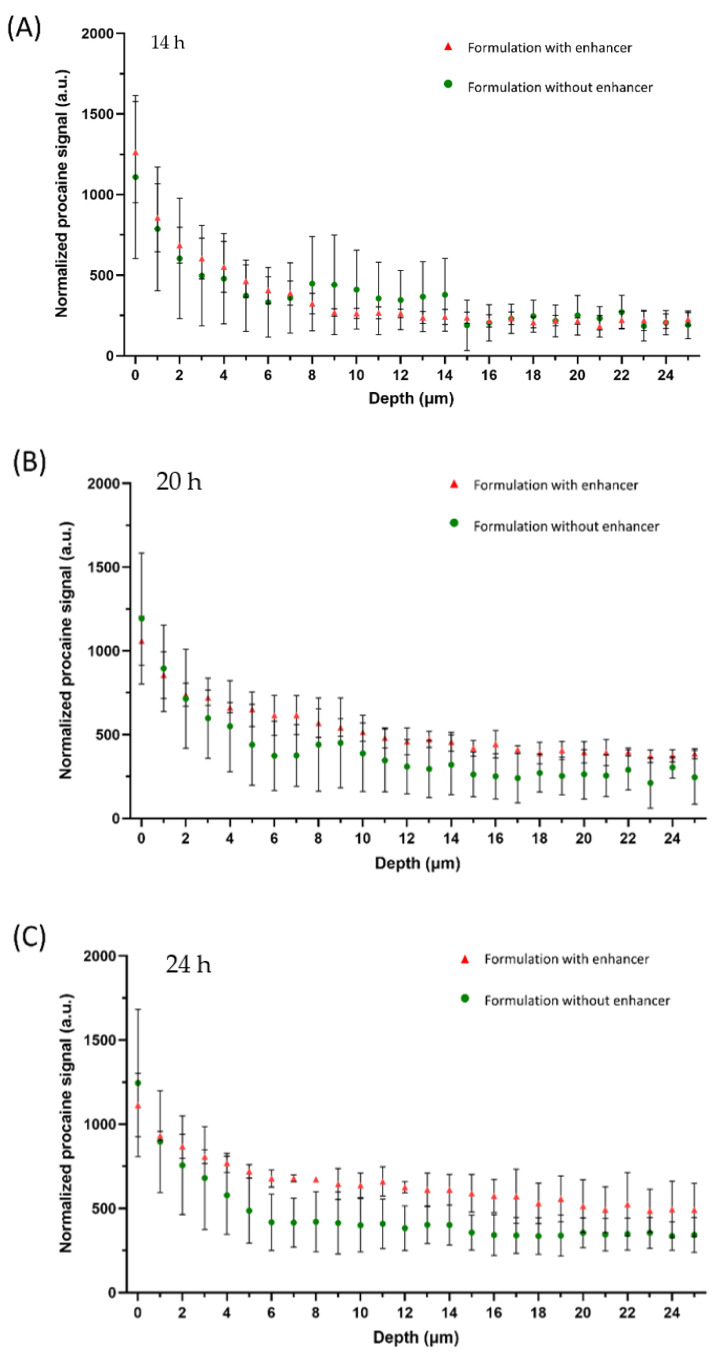
Penetration profiles of procaine HCl in porcine skin after the skin incubation time of 14 h (**A**), 20 h (**B**) and 24 h (**C**) with the measurements of the in-line device. Two procaine-HCl containing formulations were compared: formulation with enhancer (red triangles) and without enhancer (green circles). Mean ± SD (*n* ≥ 9).

Figure 5 shows the results of in-situ measurements of procaine-HCl penetration into porcine ear skin over a total incubation time of 24 h.

For both formulations, after 14 h, procaine could be found at a skin depth of more than 20 µm (Figure 5A), although most of the drug was detected within the first 10 µm of the skin. As expected, the penetration profile of the formulation containing the penetration enhancer shows a higher procaine signal within the first 7 µm of the stratum corneum.

After 20 h incubation time, the enhancement effect of L23 is clearly visible and the penetration profiles of both formulations show the same characteristics as the profiles obtained by off-line measurements (Figure 5B). Still, the highest amount of procaine was found within the first 10 µm of the stratum corneum, for both formulations. While the procaine signal of the reference formulation decreased after 5 µm, the profile of the formulation with penetration enhancer shows a flatter course, with a distinct procaine signal even below a skin depth of 20 µm. The enhancement effect was even more pronounced after 24 h. As Figure 5C shows, the amount of procaine within the stratum corneum was significantly higher after incubating with L23 as penetration enhancer. Still, the largest amount of procaine was found within a skin depth of 10 µm for both formulations, although the decrease in procaine signal was more pronounced for the reference formulation. Below a skin depth of 20 µm, procaine was detected for both formulations—the signal in both cases was considerably stronger compared to the previous time points.

### 3.3. In-Line and Off-Line Comparisons

Monitoring the in-situ depth penetration of a drug by CRS from an aqueous solution has already been performed by our group [28]. Following the depth penetration of procaine-HCl from a semisolid formulation in-situ constituted a completely new challenge. While an aqueous solution serves as a perfect immersion medium for the objective used, a hydrogel-layer between objective and skin could be used as an immersion medium too, but leads to a certain signal attenuation, which could, fortunately, be compensated by higher laser intensity. To obtain an evaluable signal, an absolute absence of air-bubbles in the hydrogel-layer is absolutely crucial. Because the formulations used in these experiments were fluid at low temperatures, a hydrogel-layer free of air inclusions could be achieved by applying the cold formulation using a pipette.

For off-line measurements, the relative amount of procaine penetration was obtained with a procaine spectral signal normalized by the amide-I signal. Although the advantages of using the phenylalanine peak for normalization and relative quantification have been well discussed [1,33], its overlap with the glass peak signal in the off-line detections precludes its use here. In this case, the normalized ratio would be still higher than 0 even without the contribution of the procaine signal, since the procaine signal was partly overlapped with the amide-I peak. Thus, by calculating the normalized ratio, with a constant value of around 0.27, as shown in Figure 4, would be the drug penetration baseline that cannot detect any procaine signals.

Despite the 14 h penetration profiles for the reference formulation, both methods lead to comparable results. Especially the depth profiles of the formulation containing the penetration enhancer show a very similar course at each time point. After 14 h, procaine was mostly located within the first 10 µm of the stratum corneum. At a skin depth below 10 µm, both formulations show the same characteristics as the course flattens. However, there was still a drug signal at 20 µm skin depth. Correlated penetration properties were also described by L. Binder et al. [5] in their interlab comparability study. As the mentioned study compared two different CRS-devices, the effect of the measurement setup used has been already described. Likewise, by calculating the AUC of varied penetration profiles, the enhancement effects can be properly compared by analyzing their significant differences.

**Figure 6:**
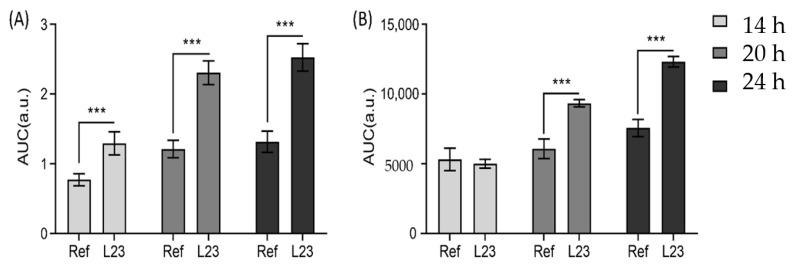
Area under the curve (AUC) comparison of off-line (**A**) and in-line (**B**) measurements between formulation with enhancers (L23) and formulation without enhancers (Ref) at different time points (14, 20 and 24 h). *** *p* < 0.001. Mean ± SD (*n* ≥ 9).

As shown in Figure 6, off-line measurements show evident enhancement effects at three incubation time points comparing two different formulations. The reason why the in-situ measurements show no significant difference between reference formulation and the formulation with penetration enhancer at the 14 h time point could be explained by the hydration state of the skin in the experimental setup, which is due to the fact that in-situ measurements were more affected by swelling of the skin during long incubation times, while skins incubated in conventional Franz-diffusion cells were patted dry before being analyzed.

Taken into account that the formulation with penetration enhancer shows a noticeably smaller standard deviation than the reference formulation, a different hydration state of the skin could be the reason for the similarity of both penetration profiles. As L23 affects the water uptake of the skin, after 14 h the stratum corneum may become saturated, whereas the skin incubated with the reference formulation still shows signs of swelling, leading to higher variability in the procaine signal. Furthermore, the effect of temperature should be considered. Although the skin in both methods was incubated at 32 °C, this temperature is kept constant for the whole in-line drug penetration monitoring process, while the off-line measurements were performed at room temperature by stopping the incubation process. This condition could lead to differences in penetration profiles especially while working with saturated solutions, that could crystalize during measurements at room temperature while being soluble at 32 °C [34,35].

Another big difference that should be taken into account is the impact of the donor animals used. As the skin samples from one donor allow for a limited number of measurements, two different donors were used in respective in-line and off-line studies, which may also lead to differences. Different donors may respond differently to the penetration enhancer [32].

After 20 h incubation time, the enhancement effect was visible in both methods and again, the penetration profiles and depths were well correlated. For both methods, a shift of the depth profiles to deeper skin regions in comparison to the 14 h time point was observed—the curves of the hydrogels with penetration enhancer show a steady decrease in signal until a skin depth of 15 µm. For the subsequent course, there is only a minimal decrease in signal.

While the off-line measurements show an enhancement effect mostly for the first 15 µm of the skin, a strong effect in deeper skin regions is visible for the in-situ measurements, especially after 24 h incubations, where the formulation with penetration enhancer shows a stronger shift of signal to deeper skin regions. The penetration profile flattens after a skin depth of 5 µm, showing an almost constant course with only a small signal decrease from 6 to 25 µm, while the off-line measurements show a constant signal decrease until a skin depth of 14 µm. As Figure 6 shows, the enhancement effect of L23 is more pronounced in the off-line measurements, but still both experimental setups lead to similar results. The differences in the enhancement effect are caused as the in-line measurements show a stronger procaine penetration out of the reference formulation.

## 4. Conclusions

In this study, we compared the results on the skin penetration of procaine-HCl obtained by two different methods, in-line and off-line measurements. Although the spectral information in both cases was recorded using the same Raman microscope, differences in skin penetration profiles were expected to vary because of the different experimental setups, in particular the incubation step and the different donor animals used. Our results show that both methods demonstrate the expected and already described influences of L23 as a penetration enhancer for procaine-HCl embedded formulations.

Furthermore, the data evaluation in both methods differs, as the phenylalanine peak used for signal-normalization in the in-line measurements was not available in the off-line measurements because a glass coverslip was needed in order to use the same objective for both experimental setups. As the penetration profiles of the formulation containing the penetration enhancer show, the different data evaluation methods led to highly comparable results.

While in-line measurements have the big advantage of being less labor-intensive and time-consuming, there are also some effects on the obtained depth profiles, such as hydration state of the skin, for example, that must be considered if using such an experimental setup. Whereas off-line measurements are more laborious, consume more resources and are, therefore, more expensive, the actual measurement could be repeated more often, as it has no need to be on time, as the in-line measurements. Because various methods and experimental setups regarding skin penetration studies are described and discussed, comparative studies are very important. As our study shows, both methods obtain comparable results.

## Figures and Tables

**Table 1:** Composition of the formulation * used (g).

Formulation	Hydroxypropyl Methylcellulose	Poloxamer 407	L23	Procaine-HCl	Water
A	0.2	2.0	0	1.0	6.8
B	0.2	2.0	0.5	1.0	6.3

* The formulations were stored at 5 °C until the day of usage.

## Data Availability

Data sharing is not applicable to this article.

## References

[1] Choe C., Choe S., Schleusener J., Lademann J., Darvin M.E. (2019). Modified normalization method in in vivo stratum corneum analysis using confocal Raman microscopy to compensate nonhomogeneous distribution of keratin. J. Raman Spectrosc..

[2] Björklund S., Engblom J., Thuresson K., Sparr E. (2010). A water gradient can be used to regulate drug transport across skin. J. Control. Release.

[3] Shakeel F., Ramadan W. (2010). Transdermal delivery of anticancer drug caffeine from water-in-oil nanoemulsions. Colloids Surfaces B Biointerfaces.

[4] Lunter D., Daniels R. Measuring skin penetration by confocal Raman microscopy (CRM): Correlation to results from conventional experiments. Proceedings of the Medical Imaging 2016: Biomedical Applications in Molecular, Structural, and Functional Imaging.

[5] Lunter D.J. (2016). How Confocal Is Confocal Raman Microspectroscopy on the Skin? Impact of Microscope Configuration and Sample Preparation on Penetration Depth Profiles. Ski. Pharmacol. Physiol..

[6] Lunter D. (2016). Determination of skin penetration profiles by confocal Raman microspectroscopy: Statistical evaluation of optimal microscope configuration. J. Raman Spectrosc..

[7] Lunter D., Daniels R. (2014). Confocal Raman microscopic investigation of the effectiveness of penetration enhancers for procaine delivery to the skin. J. Biomed. Opt..

[8] Miloudi L., Bonnier F., Tfayli A., Yvergnaux F., Byrne H.J., Chourpa I., Munnier E. (2017). Confocal Raman spectroscopic imaging for in vitro monitoring of active ingredient penetration and distribution in reconstructed human epidermis model. J. Biophotonics.

[9] Ascencio S.M., Choe C., Meinke M.C., Müller R.H., Maksimov G.V., Wigger-Alberti W., Lademann J., Darvin M.E. (2016). Confocal Raman microscopy and multivariate statistical analysis for determination of different penetration abilities of caffeine and propylene glycol applied simultaneously in a mixture on porcine skin ex vivo. Eur. J. Pharm. Biopharm..

[10] Dos Santos L., Tippavajhala V.K., Mendes T.D.O., Da Silva M.G.P., Fávero P.P., Soto C.A.T., Martin A.A. (2019). Evaluation of penetration process into young and elderly skin using confocal Raman spectroscopy. Vib. Spectrosc..

[11] Dos Santos L., Sousa M.P.J., Azoia N.G., Cavaco-Paulo A.M., Martin A.A., Favero P.P. (2017). In vivo confocal Raman spectroscopy and molecular dynamics analysis of penetration of retinyl acetate into stratum corneum. Spectrochim. Acta Part A Mol. Biomol. Spectrosc..

[12] Klang V., Schwarz J.C., Lenobel B., Nadj M., Auböck J., Wolzt M., Valenta C. (2012). In vitro vs. in vivo tape stripping: Validation of the porcine ear model and penetration assessment of novel sucrose stearate emulsions. Eur. J. Pharm. Biopharm..

[13] Jacobi U., Kaiser M., Toll R., Mangelsdorf S., Audring H., Otberg N., Sterry W., Lademann J. (2007). Porcine ear skin: An in vitro model for human skin. Ski. Res. Technol..

[14] Tfayli A., Guillard E., Manfait M., Baillet-Guffroy A. (2012). Raman spectroscopy: Feasibility of in vivo survey of stratum corneum lipids, effect of natural aging. Eur. J. Dermatol. EJD.

[15] Caspers P.J., Bruining H.A., Puppels G.J., Lucassen G.W., Carter E.A. (2001). In Vivo Confocal Raman Microspectroscopy of the Skin: Noninvasive Determination of Molecular Concentration Profiles. J. Investig. Dermatol..

[16] Albèr C., Brandner B., Björklund S., Billsten P., Corkery R., Engblom J. (2013). Effects of water gradients and use of urea on skin ultrastructure evaluated by confocal Raman microspectroscopy. Biochim. Biophys. Acta Biomembr..

[17] Ilić T., Pantelić I., Lunter D., Đorđević S., Marković B., Ranković D., Daniels R., Savić S. (2017). Critical quality attributes, in vitro release and correlated in vitro skin permeation—in vivo tape stripping collective data for demonstrating therapeutic (non)equivalence of topical semisolids: A case study of “ready-to-use” vehicles. Int. J. Pharm..

[18] Escobar-Chavez J.J., Merino-Sanjuán V., López-Cervantes M., Urban-Morlan Z., Piñón-Segundo E., Quintanar-Guerrero D., Ganem-Quintanar A. (2008). The Tape-Stripping Technique as a Method for Drug Quantification in Skin. J. Pharm. Pharm. Sci..

[19] Vyumvuhore R., Michael-Jubeli R., Verzeaux L., Boudier D., Le Guillou M., Bordes S., Libong D., Tfayli A., Manfait M., Closs B. (2018). Lipid organization in xerosis: The key of the problem?. Int. J. Cosmet. Sci..

[20] Nagelreiter C., Raffeiner S., Geyerhofer C., Klang V., Valenta C. (2013). Influence of drug content, type of semi-solid vehicle and rheological properties on the skin penetration of the model drug fludrocortisone acetate. Int. J. Pharm..

[21] Zhang Z., Lunter D. (2018). Confocal Raman microspectroscopy as an alternative to differential scanning calorimetry to detect the impact of emulsifiers and formulations on stratum corneum lipid conformation. Eur. J. Pharm. Sci..

[22] Zhang Z., Lunter D. (2018). Confocal Raman microspectroscopy as an alternative method to investigate the extraction of lipids from stratum corneum by emulsifiers and formulations. Eur. J. Pharm. Biopharm..

[23] Wolf M., Halper M., Pribyl R., Baurecht D., Valenta C. (2017). Distribution of phospholipid based formulations in the skin investigated by combined ATR-FTIR and tape stripping experiments. Int. J. Pharm..

[24] Hoppel M., Holper E., Baurecht D., Valenta C. (2015). Monitoring the Distribution of Surfactants in the Stratum Corneum by Combined ATR-FTIR and Tape-Stripping Experiments. Ski. Pharmacol. Physiol..

[25] Hoppel M., Baurecht D., Holper E., Mahrhauser D., Valenta C. (2014). Validation of the combined ATR-FTIR/tape stripping technique for monitoring the distribution of surfactants in the stratum corneum. Int. J. Pharm..

[26] Hathout R.M., Mansour S., Mortada N.D., Geneidi A.S., Guy R.H. (2010). Uptake of Microemulsion Components into the Stratum Corneum and Their Molecular Effects on Skin Barrier Function. Mol. Pharm..

[27] Belsey N.A., Garrett N.L., Contreras-Rojas L.R., Pickup-Gerlaugh A.J., Price G.J., Moger J., Guy R.H. (2014). Evaluation of drug delivery to intact and porated skin by coherent Raman scattering and fluorescence microscopies. J. Control. Release.

[28] Krombholz R., Lunter D. (2020). A New Method for In-Situ Skin Penetration Analysis by Confocal Raman Microscopy. Molecules.

[29] Shin S.-C., Cho C.-W., Yang K.-H. (2004). Development of lidocaine gels for enhanced local anesthetic action. Int. J. Pharm..

[30] Liu Y., Lunter D. (2020). Systematic Investigation of the Effect of Non-Ionic Emulsifiers on Skin by Confocal Raman Spectroscopy—A Comprehensive Lipid Analysis. Pharmaceutics.

[31] Liu Y., Lunter D.J. (2020). Tracking heavy-water-incorporated confocal Raman spectroscopy for evaluating the effects of PEGylated emulsifiers on skin barrier. J. Biophotonics.

[32] Binder L., Valenta C., Lunter D. (2020). Determination of skin penetration profiles by confocal Raman microspectroscopy: Evaluation of interindividual variability and interlab comparability. J. Raman Spectrosc..

[33] Shi L., Zheng C., Shen Y., Chen Z., Silveira E.S., Zhang L., Wei M., Liu C., De Sena-Tomas C., Targoff K. (2018). Optical imaging of metabolic dynamics in animals. Nat. Commun..

[34] Franzen L., Vidlářová L., Kostka K.-H., Schaefer U.F., Windbergs M. (2013). Freeze-drying as a preserving preparation technique forin vitrotesting of human skin. Exp. Dermatol..

[35] Goh C.F., Craig D.Q.M., Hadgraft J., Lane M.E. (2017). The application of ATR-FTIR spectroscopy and multivariate data analysis to study drug crystallisation in the stratum corneum. Eur. J. Pharm. Biopharm..

